# Molecularly Imprinted Methyl-Modified Hollow TiO_2_ Microspheres

**DOI:** 10.3390/molecules27238510

**Published:** 2022-12-03

**Authors:** Vanessa R. A. Ferreira, Manuel A. Azenha, Carlos M. Pereira, António F. Silva

**Affiliations:** CIQ-UP, Institute of Molecular Sciences, Departamento de Química e Bioquímica, Faculdade de Ciências da Universidade do Porto, Rua do Campo Alegre, 4169-007 Porto, Portugal

**Keywords:** methyl-modified, hollow TiO_2_ microspheres, selectivity, molecular imprinting, sol-gel

## Abstract

The possibility of generating organically modified hollow TiO_2_ microspheres via a simple sol-gel synthesis was demonstrated for the first time in this work. A mixture of titania precursors, including an organically modified precursor, was used to obtain methyl-modified hollow TiO_2_ microspheres selective for bilirubin by the molecular imprinting technique (Methyl-HTM-MIM). Methyl-HTM-MIM were prepared by a sol-gel method using titanium (IV) isopropoxide (TTIP), and methyltitanium triisopropoxide (MTTIP) as precursors. Two ratios of titania precursors were tested (1/6 and 1/30 mol_MTTIP_/mol_TTIP_). With the characterization results obtained by the SEM and ATR-FTIR techniques, it was possible to establish that only the 1/30 mol_MTTIP_/mol_TTIP_ ratio allowed for the preparation of hollow spheres with a reasonably homogeneous methylated-TiO_2_ shell. It was possible to obtain a certain degree of organization of the hybrid network, which increased with calcination temperatures. By adjusting isothermal adsorption models, imprinting parameters were determined, indicating that the new methylated microspheres presented greater selectivity for bilirubin than the totally inorganic hollow TiO_2_ microspheres. The effectiveness of the molecular imprinting technique was proven for the first time in an organically modified titania material, with imprinting factor values greater than 1.4, corresponding to a significant increase in the maximum adsorption capacity of the template represented by the molecularly imprinted microspheres. In summary, the results obtained with the new methyl-HTM-MIM open the possibility of exploring the application of these microspheres for selective sorption (separation or sensing, for example) or perhaps even for selective photocatalysis, particularly for the degradation of organic compounds.

## 1. Introduction

The production of organically modified TiO_2_-based materials may be an emerging area of interest, similarly to the widespread use of organically modified silanes (ORMOSILs) to produce hybrid organic–inorganic silicas [1,2]. However, the hybrids to which we refer are those in which the organic groups are covalently integrated into the network by a normal sol-gel process (polycondensation) through the use of a R-Ti(OR’)_3_ precursor, where R represents a non-hydrolysable group. These should not be confounded with composite materials obtained by mixing, doping, or impregnating TiO_2_ networks with organic molecules or particles (e.g., [3,4,5]).

The use of organically modified metal alkoxides has been described for the preparation of organically modified TiO_2_-based materials [6]. However, this has been a rarely used option, possibly owing to the inherent difficulty in understanding the chemistry of organically modified titania alkoxide precursors, which is much more exigent compared to ORMOSILs [7]. Thull et al. [8] described the synthesis of copolymers based on TiO_2_, with a hybrid organic/inorganic network obtained by polymerization of titanium alkoxides modified with polymerizable organic ligands. Sethi and collaborators [9] described the preparation of organically modified titania nanoparticles for drug delivery by oil-in-water microemulsion using the organometallic precursor cyclopentadienyl titanium trichloride. According to the authors, the presence of organic constituents in the inorganic matrix allowed for reconciliation of flexibility, porosity, and ability to host bioactive molecules, as well as their subsequent release. According to literature data, some organically modified titania alkoxides stand out as more stable and even less corrosive and ecotoxic than standard titania alkoxides [6]. These characteristics are the result of the special bond between the organofunctional group and the metal, which also interferes in the behavior of hydrolysis and consequent gelation of new compounds with hybrid characteristics [8]. The use of these organically modified titania precursors may be necessary to functionalize the surfaces of various purely inorganic materials, further increasing the recent interest in the development of molecular imprinting materials based on TiO_2_ [10,11,12]. This is an idea that has already been widely explored in the area of sol-gel molecular imprinting with ORMOSILs [13]. The exploitation of these organosilane precursors to obtain hybrid inorganic/organic molecularly imprinted materials has proven the possibility of obtaining materials with various characteristics in terms of the chemical composition of the precursors and the proportion of inorganic and organic components. These superficial differences have already been widely discussed in the literature as enhancers of the effectiveness of the molecular imprinting technique [14,15,16,17].

Recently, there have been reports on the application of molecular imprinting technology in combination with hollow TiO_2_ materials in non-spherical format [18,19]. Molecular imprinting allows for the nanostructuring of materials with special cavities or receptor sites with an optimized configuration for the adsorption of a structure of interest. However, to date, only two studies have demonstrated the possibility of reconciling photonic efficiency associated with the hollow spherical shape and selectivity using the molecular imprinting technique [20,21]. These two studies proved the possibility of molecular imprinting directly on the TiO_2_ shell with its hollowness and crystallinity, in order to produce highly effective and bilirubin-selective photocatalysts. Accordingly, in the present study, for comparison of imprinting features, bilirubin was selected as the template. Protoporphyrin was selected as an analogous compound for comparison and confirmation of the selectivity of the molecular imprinting technique, as in previous studies.

Therefore, the present work was designed to test, for the first time, the use of a mixture of precursors, including an organically modified precursor, to obtain organically modified hollow TiO_2_ microspheres selective for bilirubin by molecular imprinting technique (methyl-HTM-MIM). Of the commercially available organically modified titania precursors described in the literature, were initially considered those with small and simple organic groups, which would possibly cause less disruption in the formation of the oxometallic network and which would possibly increase interactions with the template to be tested (bilirubin), namely methyltitanium triisopropoxide (MTTIP), and phenyl titanium triisopropoxide. The two precursors have been described in the literature as mediators of chemical reactions, such as cyclopropanation [22,23,24,25,26,27] and catalytic reactions [28] (where metal and functional group interactions are discussed). There are not literature reports describing the use of any of these precursors for the synthesis of hybrid-HTM nor hybrid-HTM-MIM.

Herein we used a mixture of titanium (IV) isopropoxide (TTIP), purely inorganic, and MTTIP precursors. The latter was included, given its low cost and its component organic group, i.e., a non-polar group. Furthermore, we expected that the methyl groups would minimally interfere in the desired oxometallic network.

The new photocatalysts developed in this work were characterized, and their adsorption properties were studied by fitting to isothermal models. The possibility of reconciling the presence of organic groups with the calcination process at mild temperatures was also tested in order to obtain some degree of organization in the TiO_2_ shell structure to promote increased photocatalytic efficiency.

The microspheres are designated in this work by a code of as many as five components separated by a dash. The component “methyl” is the first to appear for organically modified microspheres; the next component represents the morphology of the spheres, i.e., hollow (H) or core–shell (C); the next component indicates molecularly imprinted material (MIM) or non-molecularly imprinted material (NIM); the next component appears only when acidic treatment was carried out before calcination (HCl); and the last component indicates whether the material has been calcined and, if so, the calcination temperature in °C, i.e., 200, 250, or 500. For example, methyl-H-MIM-HCl-200 represents the imprinted methyl-modified hollow microspheres obtained with acidic pretreatment before calcination at 200 °C, whereas methyl-H-MIM-200 designates the same material but without being subjected to acidic pretreatment, and methyl-H-MIM designates the same material but without acidic pretreatment or calcination.

## 2. Results and Discussion

### 2.1. Morphological and Compositional Properties of the Microspheres 

**Figure 1:**
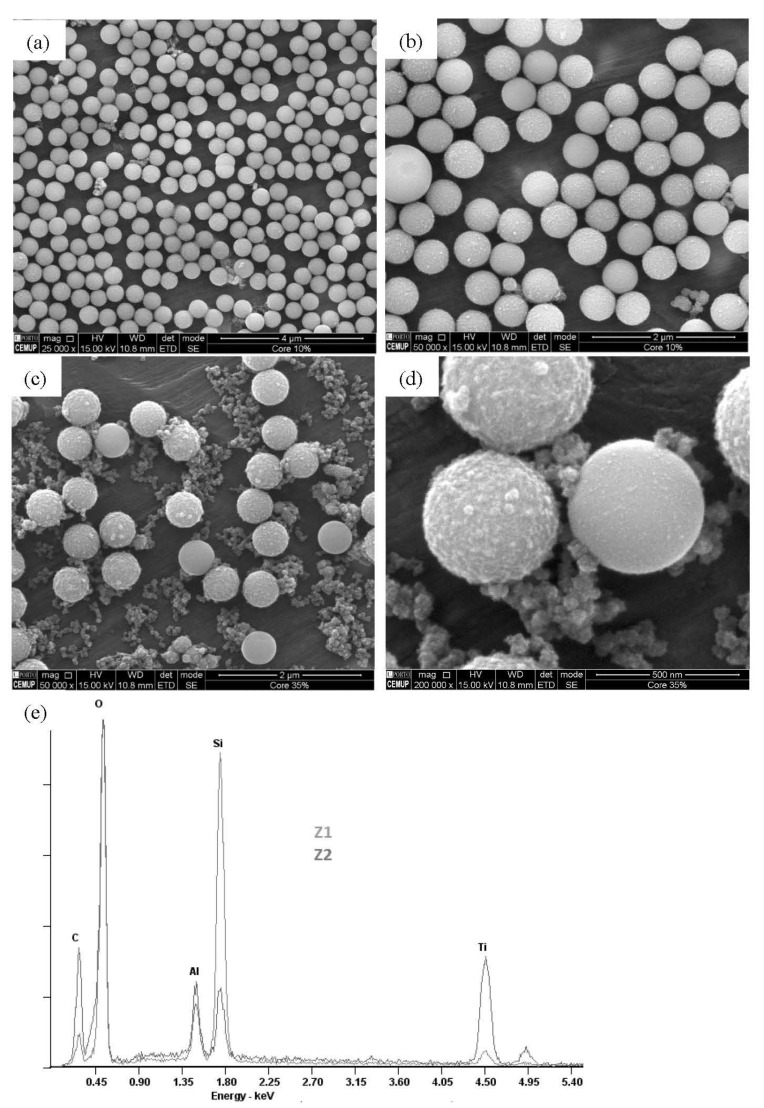
Representative SEM micrographs of methyl-C-MIM microspheres (SEM images (**a**,**b**) correspond to 1/30 mol_MTTIP_/mol_TTIP_, and the other SEM-EDS images correspond to 1/6 mol_MTTIP_/mol_TTIP_ (**c**,**d**,**e**).

In general, the SEM images presented in Figure 1 confirm the microspherical shape of the materials obtained with the new synthesis method containing a mixture of titania precursors. In contrast to what was observed in totally inorganic TiO_2_ hollow microspheres [20], a characteristic superficial roughness of the new organically modified microspheres was also detected. This roughness is more evident in the microspheres obtained with a greater amount of MTTIP, as shown in Figure 1c,d.

In addition to the microspheres, irregularly shaped nanoparticles were detected, which, according to an EDS analysis, contain the elements Ti, C, and O (Figure 1d,e). The greater the proportion of MTTIP used in the synthesis, the greater the amount of irregular nanoparticles and their carbon content (Figure 1c,d), which may reflect the formation of methyl-rich sol-gel aggregates favored by an increased abundance of the organically modified titania precursor. The presence of a methyl group on the surface of methyl-C-MIM was confirmed by EDS data, as shown in Figure 1d. The area marked with a red square is a methyl-modified TiO_2_ layer. In contrast, the zone marked with a green square, corresponding to irregular particles, contains only Ti, C, and O elements. The high intensity of the characteristic band of carbon reveals the preferential condensation from the methyl-modified precursor.

A proportion of 1/30 mol_MTTIP_/mol_TTIP_ proved to be the most suitable for obtaining organically modified microspheres, with characteristic roughness derived from the presence of methyl groups on the surface. The same conditions led to fewer irregular particles as a consequence of the hydrolysis of excess precursors, namely MTTIP.

**Figure 2:**
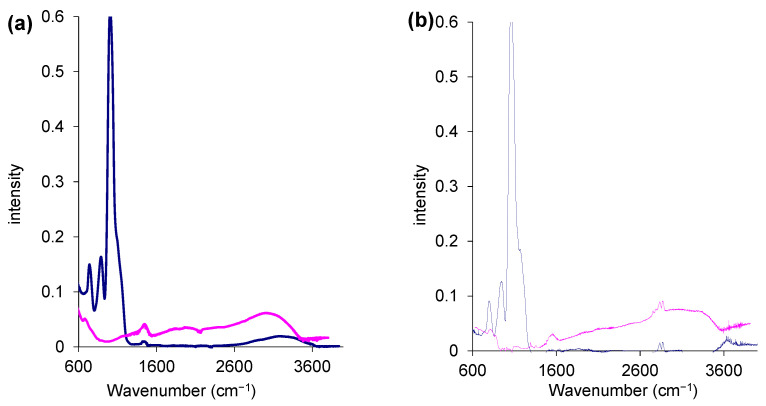
ATR-FTIR spectra of methyl-C-MIM (●●●●) and Methyl-H-MIM (●●●●) with (**a**) 1/30 mol_MTTIP_/mol_TTIP_; and (**b**) 1/6 mol_MTTIP_/mol_TTIP_.

Figure 2 shows the FTIR spectra of methyl-C-MIM and methyl-H-MIM obtained at both MTTIP/TTIP ratios. The profiles observed in the spectra are similar to those previously discussed in the corresponding unmodified hollow TiO_2_ microspheres (c.f., [20] for a more detailed peak assignment of the bands). In the case of methyl-H-MIM microspheres (Figure 2 pink spectra), the most intense band occurs at ≈950 cm^−1^ (Ti-O stretching), which was also detected in the spectra obtained from the analysis of hollow microspheres obtained using only a TTIP precursor [20] and solid TiO_2_ microspheres [29]. In the case of methyl-C-MIM microspores (Figure 2, blue spectrum) the results are similar to those obtained with the same analog microspheres synthesized with TTIP precursor only [20], for which the Si-O stretching at ~1100 cm^−1^ was the dominant band.

The C-H flexural vibration bands at 1480 cm^−1^, as well as the bands at 2924 and 2850 cm^−1^, can be attributed to the stretching vibrations of CH_2_ and CH_3_ and may be an indication of the presence of the methyl group added to the surface of the methyl-C-MIM microspheres. The bands at 2924 and 2850 cm^−1^ were detected only in the spectra of the microspheres synthesized with a mixture of precursors in a proportion of 1/6 (mol_MTTIP_/mol_TTIP_). 

In general, the collected spectra indicate of the effectiveness of the core removal procedure and enable identifying the presence of a methyl group on the surface of the microspheres obtained with a mixture of titania precursors.

With the aim of obtaining a compromise between increased affinity to the organic template compound by adding organic groups to the surface of the microspheres with a hollow shape and eventually achieving some degree of crystallinity of the TiO_2_ shell, which might be advantageous for eventual photocatalytic applications, the newly pre-pared methyl-H-MIM-HCl was subjected to calcination at varying temperatures and for varying times (500 °C, 2 h; 250 °C, 17 h; 200 °C, 72 h; and 150 °C, 7 days). These calcination conditions were selected according to our previous research [21]. Concretely, calcination at 200 and 250 °C allowed us to obtain hollow TiO_2_ microspheres with high crystallinity as an anatase. However, the existence of organic groups in the TiO_2_ network does not allow for a pure crystalline phase of TiO_2_ because the presence of a methyl group in the oxometallic network interferes with the periodic arrangement of Ti and O atoms necessary for pure crystalline phases known to exist for TiO_2_ materials (anatase, rutile, and brookite). Thus, only a limited level of organization of the TiO_2_ network promoted by the calcination effect can be expected. Nevertheless, it would constitute a positive evolution from the initial amorphous state of titania. Mild calcination temperatures were tested in order to maintain the synergism between the presence of organic groups within an imprinted TiO_2_ network but with some degree of structural organization, with possible crystalline microregions (grains or crystallites) possibly separated by crystallographic defects associated with the methyl group. These characteristics of crystalline microphases associated with some structural order may increase the efficiency of the photonic response of these materials.

**Figure 3:**
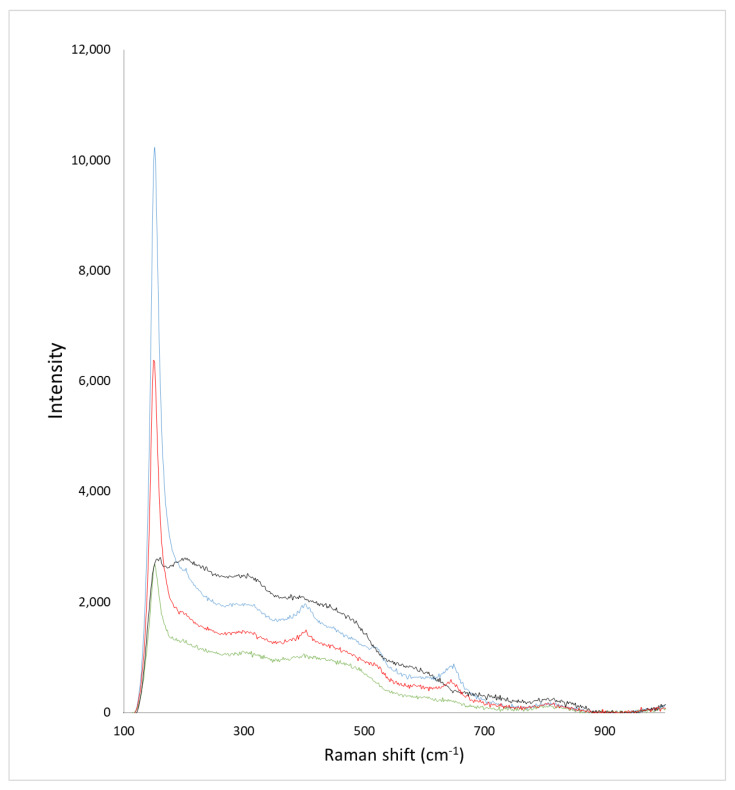
Raman spectra of methyl-H-MIM-HCl-500 (●●●●), methyl-H-MIM-HCl-250 (●●●●), methyl-H-MIM-HCl-200 (●●●●), and methyl-H-MIM-HCl-150 (●●●●) with 1/30 mol_MTTIP_/mol_TTIP_.

Analysis of the Raman spectra obtained for the methyl-H-MIM-HCl-xxx microspheres (Figure 3) shows that the characteristic profile of the anatase phase was not obtained under any of the temperature/time conditions. A comparison of a Raman spectrum of a sample containing highly crystalline anatase [21] with spectra obtained with methyl-H-MIM-HCl-xxx reveals representative peaks of anatase at 150, 400, and 690 cm^−1^, indicating the presence of crystalline microphases in the microspheres. Higher crystallinity could not be obtained even with 2 h calcination at 500 °C. These results are possibly related to a change in the structural organization obtained in the presence of methyl groups, which, even after their thermal decomposition, prevented the structural rearrangement necessary to obtain a highly crystalline network.

Taking into account the obtained results, the remaining experiments were carried out with calcined (250 °C, 200 °C, and 150 °C) or uncalcined methyl-modified TiO_2_ microspheres with 1/30 mol_MTTIP_/mol_Ti_.

### 2.2. Textural Properties of the Microspheres 

As shown in Table 1, the methyl-C-MIM/Methyl-C-NIM and methyl-H-MIM/Methyl-H-NIM pairs presented the optimal textural features. These microspheres had high values of surface area/shell thickness (0.47 to 1.73 m^2^/g/nm) as a result of the high pore volume/shell thickness (1.3 to 3.9 × 10^3^.cm^3^/g/nm) compared to all other microspheres. 

In agreement with the literature [30,31,32,33], the results in Table 1 show that the MIM has higher surface area/thickness than the respective NIM, consistent with the higher surface area/thickness ratio resulting the high MIM pore volumes and consequent surface area compared to the corresponding NIM. However, the opposite has also been described in cases of successful molecular imprinting [20,21,34,35], meaning that the sorption features of MIM are mostly governed by the quality and quantity of the recognition sites.

The abovementioned observation concerning MIM’s increased surface area was more evident in methyl-modified core–shell microspheres compared to the hollow analogs, despite the pore volume being, in general, higher in hollow microspheres. These results are possibly the result of the pore size being affected when the microspheres were subjected to SiO_2_ removal. As shown in Table 1, the pore size is smaller for the methyl-C-MIM than for methyl-C-NIM. Consequently, higher surface area/thickness and larger pore size were determined for methyl-C-MIM and methyl-C-NIM compared with the respective methyl-H-MIM and methyl-H-NIM. However, an exception was observed corresponding to the pore size of the methyl-H-MIM-HCl (4.3 nm), which is larger than that of methyl-C-MIM-HCl (3.8 nm) and increased compared to the analog methyl-H-MIM (3.8 nm). These results indicate that the acidic treatment allowed the microspheres to maintain the integrity of the original pores after removing the core with etching solution (NaOH 2.5 mol/L), as the same behavior was not observed in the case of methyl-C-MIM.

For methyl-H-MIM-HCl-xxx, regardless of the temperatures used, the values of pore size, pore volume, and consequent surface were reduced in all microspheres. The most evident change was in methyl-H-MIM-HCl, which, after calcination, became microporous at all calcination temperatures, whereas the respective NIM maintained mesoporous characteristics but with a pore size reduction of approx. 1 nm. The acidic treatment enabled the maintenance of the integrity of the mesopores during core removal but was not effective in preventing the pores from collapsing with an increase in temperature, as previously verified in [21]. This suggests that the presence of the template associated with the presence of a methyl group on the surface of the microspheres made the 3D Ti-O-Ti network less stable to temperature causing the collapse of a large portion of the pores.

The same effect was not verified for methyl-C-MIM-HCl, possibly because it maintained a core, which promoted stability during the calcination process, regardless of the temperature used, partially maintaining the surface characteristics.

A comparison of the new methyl-modified microspheres with the corresponding unmodified microspheres [20] shows that the surface area/thickness of methyl-H-MIM and methyl-C-MIM (0.5–1.7 m^2^/g/nm) is lower than the obtained with only one precursor (1.8–3.4 m^2^/g/nm), although the pore volume/thickness ratio is higher in methyl-H-MIM and methyl-C-MIM (1.3 to 3.9 × 10^3^.cm^3^/g/nm) compared with the unmodified analogs (0.6 to 0.7 × 10^3^.cm^3^/g/nm). A similar comparison can be made between the methyl-H-MIM-HCl-xxx and methyl-C-MIM-HCl-xxx microspheres, with the unmodified microspheres described by Ferreira et al. [21], with an even greater reduction in the surface area/thickness (0.9–2.3 m^2^/g/nm [21] to 0.08–0.13 m^2^/g/nm). This possibly occurs because the desired effect of protecting the integrity of the pores with the previous acidic treatment was not effective in the oxometallic network containing the methyl group, causing a drastic reduction in pore size.

Considering the mechanism by which the acidic treatment allows for maintenance of the integrity of the TiO_2_ shell of the hollow microspheres, there is currently no feasible explanation for its ineffectiveness in the presence of methyl groups on the surface.

### 2.3. Sorptive Features of Microspheres 

**Figure 4:**
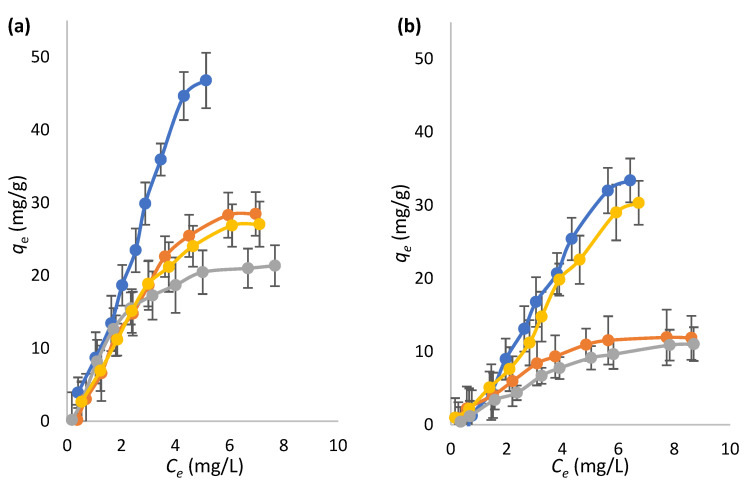
Equilibrium binding isotherms for the sorption of bilirubin (**a**) and protoporphyrin (**b**) by (●) methyl-H-MIM-HCl-200, (●) methyl-H-NIM-HCl-200, (●) methyl-H-MIM, and (●) methyl-H-NIM. Error bars represent the standard deviation of the mean result (*n* = 3).

Analysis of the isotherms shown in Figure 4 obtained with the new methyl-H-MIM and methyl-H-NIM, in comparison with their unmodified analogs [20], shows that the maximum adsorbed amounts of both bilirubin and protoporphyrin are higher with methyl-H-MIM. Further results are presented in Figure S1. It is clear that the presence of the methyl group of MTTIP on the surface of the new methyl-HTM promotes an increased ability to interact with organic compounds, namely bilirubin and protoporphyrin.

The desired molecular imprinting effect was thus verified in methyl-H-MIM-HCl-200 and methyl-H-MIM. Because an increase in the maximum adsorption capacity (*q_max_*) of bilirubin was confirmed in methyl-H-MIM and methyl-H-NIM-HCl-200 compared to the corresponding NIM, in addition to the evident selectivity of the MIM for bilirubin, it is therefore possible to conclude that the desired molecular imprinting was obtained in methyl-H-MIM-HCl-200 and methyl-H-NIM-HCl-200.

Of all methyl-modified microspheres tested, those that stood out the most were methyl-H-MIM microspheres, with a *q_max_* of approximately 47 mg/g, which is much higher than that of all others tested microspheres (*q_max_* < 29 mg/g). These results correspond with the collapse of pores resulting from the calcination process, as demonstrated by the textural analysis. The decrease in the average pore size reduced the surface area and disturbed the nanostructuration induced by the imprinted sites, thus decreasing the adsorption capacity. However, for methyl-H-MIM-HCl-xxx microspheres, the effect of molecular imprinting was not verified, with values of *q_max_* for bilirubin statistically identical for the MIM and respective NIM.

A comparing of the results of methyl-H-MIM with the unmodified analogous microspheres [20] revealed that the mixture of precursors significantly increased the *q_max_* of bilirubin, especially in the case of MIM (methyl-H-MIM = 47 mg/g; unmodified analogous microspheres [20] = 19.2 mg/g), supporting the benefit of available organic groups on the surface of methyl-H-MIM. However, a comparison of the results of methyl-H-MIM-HCl-200 and methyl-H-NIM-HCl-200 with the analogous microspheres described by Ferreira et al. [21] shows a significant reduction in the *q_max_* of bilirubin, especially in molecularly imprinted microspheres (methyl-H-MIM-HCl-200, 46.8 mg/g; unmodified microspheres, 65.0 mg/g). These results are possibly the result of the degradation of methyl groups as a result of calcination, which is associated with the collapse of the pores and the disruption of imprinted site nanostructuration, which significantly reduced the surface area/thickness and therefore the adsorption capacity.

For an improved understanding of the interference caused by calcination and acidic treatment in the molecular imprinting process of the new methyl-modified TiO_2_ microspheres, adsorption isotherm data (*qe*; *Ce*) were fitted to various isothermal models, such as the Langmuir, Freundlich, Langmuir–Freundlich (L-F) hybrid, and Hill Equation (H) (Equation (4)) models by nonlinear regression analysis. The best fits (*χ^2^* < 10^−7^) were obtained with the L-F and H models. That corresponding to MIM had a better fit to the H model, whereas NIM showed better fits to the L-F model. The sigmoidal shape observed in the MIM isotherms may be related to cooperative phenomena facilitating binding to the sorbent (positive cooperative binding, *n* ≥ 1) or hampered (negative cooperative binding *n* < 1) by previously adsorbed ligands. Fitting to the H model has been reported for MIM, namely MIM selective for, enzymes, and drugs [35,36,37,38]. 

The values of *n* obtained with the H model were always greater than 1 (1.3–2.9), indicating a positive cooperative binding mechanism associated with all studied MIM. A possible mechanism to explain the cooperativity in MIM is the existence of selective pores resulting from the imprinting of template dimers/trimers. In this case, the binding is maximized only as the cavity becomes fully populated [39]. Another possible explanation for the cooperative effect of MIM is the occurrence of an induced adjustment capacity. This refers to a process whereby the initial interaction between the material and the guest is relatively weak but induces conformational changes in the structure of the material that strengthen the bond of subsequent guest molecules [40]. Further elucidation of this question is beyond the scope of this study and requires further focused work. The result is probably due to the presence of the methyl group, which provided increased flexibility to the TiO_2_ shell. As discussed above, the presence of methyl groups does not allow for a pure crystalline organization of the TiO_2_ network, increasing it flexibility and capacity to adapt to new conformations. That may be on the basis of the cooperative effect observed during the interaction with the sorbate molecules.

Table 2 contains the data extracted by fitting to the H and L-F models (*K* and *q_max_*), as well as the molecular imprinting parameters (*IF* and *α*) determined according to *q_max_*. The values of *IF* and *α*, as a function of *K*, were not determined, as they represent different units depending on the isothermal model used for determination; therefore, they are not directly comparable.

All methyl-C-MIM and methyl-H-MIM exhibited features corresponding to a successful imprinting process, in the sense that for all groups tested, the MIM showed greater binding strength and binding capacity for bilirubin compared to NIM, leading to *IF*(*q_max_*) > 1. Furthermore, all MIM showed *K* values for bilirubin higher than the K values obtained for protoporphyrin, indicating the greater affinity of the existing pores for the template used during the synthesis process.

Although calcination negatively affected the adsorption capacity of all organically modified TiO_2_ microspheres, it favored the bilirubin selectivity of Methyl-H-MIM-HCl-xxx. As shown in Table 2, the ratio between the adsorption capacity of bilirubin and protoporphyrin was approximately twofold in the cases of methyl-H-MIM-HCl-xxx, regardless of the temperature. Thus, the *α*(*q_max_*) increased significantly (*α*(*q_max_*) ≥ 1.6), especially for MIM (*α*(*q_max_*) ≥ 1.7). However, the selectivity observed for the calcined NIMs, although slightly lower, was similar to that exhibited by the MIM, indicating that the causes of the selectivity improvement are not imprinting-related but governed by the calcination process only.

A comparison of the *α*(*q_max_*) results of methyl-H-MIM-HCl-xxx (1.6 < *α* < 1.9) with those of unmodified analogs (9.2 < *α* < 13.5 [21]) confirms a very high reduction rate in α values, probably due to the disruption of the imprinted cavities during calcination. The drastic reduction in the volume and pore size verified in the new methyl-H-MIM-HCl-xxx indicates that the acidic treatment prior to calcination did not have the desired effect of protecting the integrity of the porous network and imprinted sites.

In brief, the results obtained with the new methyl-H-MIM indicate a certain degree of order in the structure of the TiO_2_ network upon mild calcination temperatures, which is associated with the observed imprinting effect, as expressed by both sorption capacity and selectivity, opening the possibility of exploring the application of these microspheres in selective photocatalysis, namely for degradation of organic compounds.

## 3. Materials and Methods

### 3.1. Materials and Reagents

All chemicals were obtained from commercial suppliers and were used without further purification. IGEPAL CA-630, tetramethyl orthosilicate (TEOS), and ammonium citrate tribasic anhydrous from Sigma–Aldrich (purity > 99%, Darmstadt, Germany) were used to prepare the SiO_2_ microspheres. TiO_2_ precursors TTIP (purity, 98%) and MTTIP (1 mol/L in THF) were obtained from Acros and used to prepare the core–shell microspheres. Sodium hydroxide (NaOH) purchased from Fluka (purity > 98%, St.Gallen, Switzerland) was used to remove the core, producing hollow microspheres. Hydrochloric acid (HCl) obtained from Merck (37% *w*/*w*, Darmstadt, Germany) was used for acid treatment after synthesis. Organic solvents, ethanol, and isopropanol (analytical grade, Fluka) were used for synthesis. All aqueous solutions were prepared with deionized ultrapure water (Milli-Q Water Purification, Burlington, MS).

### 3.2. Methods

#### 3.2.1. Synthesis of Methyl-Modified Hollow TiO_2_ Microspheres

The synthesis method used for the preparation of the new methyl-H microspheres was the same as that described in [10], with the adaptation of the mixture of two titania precursors (TTIP/MTTIP).

The same SiO_2_ spheres were synthesized through an adaptation of the Stöber method and used as cores to obtain methyl-HTM. SiO_2_ spheres (250 mg) were dispersed in ethanol (40 mL) containing IGEPAL (2 mL) and a small amount of water (0.5 mL), and a solution containing the TTIP and MTTIP precursors in the proportions of 1/6 and 1/30 mol_MTTIP_/mol_TTIP_, respectively, was added drop-by-drop. After 2 h of reaction at room temperature, organically modified core–shell TiO_2_ microspheres (Methyl-C) were obtained. For the formation of methyl-H microspheres, samples were washed with a solution of 2.5 mol/L of NaOH (1 mL per 10 mg microspheres) for 1 day while stirring and with periodic refreshing of the washing solution.

For the preparation of methyl-H-MIM, the sane synthesis method was used, with the addition of bilirubin in a proportion of 1/100 mol_bilirubin_/mol_Ti_ to the solution containing the mixture of the titania precursors. The remainder of the synthesis process was the same, including the template removal process, which was performed simultaneously with SiO_2_ core removal during washing with 2.5 mol/L NaOH solution.

The new methyl-H-MIM and methyl-H-NIM were subjected to an acidic treatment (methyl-H-MIM-HCl and methyl-H-NIM-HCl). The acidic treatment consisted of mixing microspheres with an aqueous HCl solution (0.1 mol/L) in a proportion of 10 mg_microspheres_/mL_HCl_ under stirring for 30 min. Finally, the microspheres were subjected to calcination at 150–500 °C for 2 h–7 days, depending on the temperature.

#### 3.2.2. Characterization of the Composites and Batch Sorption Studies: Isotherms and Respective Data Treatment

Characterization methods were applied (i.e., SEM, DLS, ATR-FTIR, and N_2_ adsorption techniques), and isothermal tests were performed according the same procedures described in [20]. Raman spectra were recorded with a Ramos PA532 Ostec Raman spectrometer (Moscow, Russia), using a 532 nm excitation wavelength for 87 s.

The absorption spectra of bilirubin and protoporphyrin were examined in the wavelength range of 200 to 600 nm, using an ultraviolet-visible (UV-Vis) spectrophotometer (T60, PG instruments, Leicestershire, UK).

Theoretical isotherms were theoretically fit to experimental data using the IGOR PRO software Package. In this study, three models, i.e., the Langmuir (Equation (1)), Freundlich (Equation (2)) and Langmuir–Freundlich isotherms (L-F) (Equation (3)), were initially tested to describe the sorption of the metallic cations.

(1) $$q_{e}=\frac{q_{max}KC}{1+KC}$$

(2) $$q_{e}=\frac{q_{max}KC}{1+KC}$$

(3) $$q_{e}=\frac{q_{max}{\left(KC\right)}^{m}}{1+{\left(KC\right)}^{m}}$$

where *q_e_* corresponds to the sorbed concentration in equilibrium (mg/g), *C* is the equilibrium concentration in solution (mg/L), and *K* is a fitting constant. The constant is related to the binding affinity, and the parameter *m* refers to the heterogeneity index; its value ranges from 0 to 1, increasing with decreased heterogeneity. The Langmuir model assumes that one class of site is present on the surface, with a saturation capacity (*q_max_*) to form a complete monolayer on the surface with dissociation constant *K* (L/mg). The Freundlich isotherm, on the other hand, assumes sites with a Gaussian distribution of binding strengths. Here, the width of the Gaussian distribution describes the degree of heterogeneity through the index *m*. The difference between the L-F model and the Freundlich model is evident at high sorbate concentrations, for which the L-F model is able to represent the saturation behavior. At low sorbate concentrations, the L-F equation is reduced to the classical Freundlich equation.

On the other hand, if m approaches unity, it is indicative of a completely homogeneous sorbent surface (i.e., energetic equivalence of all binding sites); the L-F equation reduces to the classical Langmuir equation. Thus, the hybridized L-F isotherm is able to model the adsorption of solutes at high and low concentrations on both homogeneous and heterogeneous sorbents.

An additional model, the Hill isotherm model (H), was also considered. This model describes the binding of different species onto homogeneous substrates and assumes that adsorption is a cooperative phenomenon, with adsorbates at one site of the adsorbent influencing other binding sites on the same adsorbent.

The following equation represents the isothermal model:

(4) $$q_{e}=\frac{q_{max}C_{e}^{n}}{K_{d}C_{e}^{n}}$$

where *q_max_* corresponds to the maximum adsorption capacity (mg/g), *n* corresponds to the Hill cooperative coefficient, and *K_d_* is the constant dissociation (units: (mol/L)^n^).

## 4. Conclusions

In this work, the possibility of incorporating organic groups into inorganic hollow TiO_2_ microspheres was demonstrated for the first time, using two titania precursors, one of which contains a non-hydrolysable methyl group (MTTIP). A proportion of 1/30 mol_MTTIP_/mol_TTIP_ proved to be suitable for obtaining organically modified microspheres, with a SEM-observed characteristic roughness representative of the presence of methyl groups on the surface and with few irregular particles resulting from the preferential co-condensation of MTTIP.

It is possible to obtain a degree of TiO_2_ network organization, with network organization increasing with the calcination temperature. However, highly crystalline TiO_2_ may not be obtainable due to the presence of methyl groups within the material.

For the methyl-modified microspheres, the isothermal models that best-fit the obtained results were applied, i.e., the H model for the results with the MIM and the L-F model for the results of the NIM. The H model indicates a positive cooperative effect between the adsorbed molecules, resulting from the presence of methyl groups on the surface of the microspheres associated with the presence of selective pores.

The success of the molecular imprinting technique was confirmed for the first time in organically modified TiO_2_ hollow microspheres, with an increase in bilirubin adsorption capacity compared to unmodified hollow microspheres (*IF*(*q_max_*) ≥ 1.4). The higher affinity of the selective pores displayed on the surface of the organically modified molecularly imprinted microspheres for bilirubin (*K*_bilirubin_ > *K*_protoporphyrin_) was also verified. Furthermore, the calcination process evidenced the bilirubin selectivity of the methyl-H-MIM-HCl-xxx, with *α*(*q_max_*) values ≥ 1.7. These microspheres may be useful in applications that would benefit from selective sorption, such as separation or sensing applications.

Despite the difficulty in reconciling the presence of functional groups with the crystallinity of TiO_2_, an important parameter for the photocatalytic efficiency, the new methyl-H-MIM could represent a novel research direction in the area of photocatalysts, especially for use in selective photocatalysis of organic compounds. Future work, should include photocatalysis experiments to test such potential.

## Figures and Tables

**Table 1:** Microstructural properties of the methyl-modified TiO_2_ microspheres.

Methyl-Modified TiO_2_ Microspheres	Nitrogen Adsorption Technique		
	* Surface Area/Thickness (m^2^/g/nm)	** Average Pore Size (nm)	** Pore Volume/Thickness (10^3^.cm^3^/g/nm)
Methyl-C-MIM	1.73	4.6	2.0
Methyl-C-NIM	1.27	4.8	1.3
Methyl-H-MIM	1.25	3.8	3.9
Methyl-H-NIM	0.47	3.2	1.9
Methyl-C-HCl-MIM	1.55	3.8	2.2
Methyl-C-HCl-NIM	1.04	4.2	0.9
Methyl-H-HCl-MIM	0.85	4.3	2.7
Methyl-H-HCl-NIM	0.37	4.8	1.8
Methyl-C-HCl-MIM-250	1.51	4.0	1.0
Methyl-C-HCl-NIM-250	0.91	3.3	1.4
Methyl-H-HCl-MIM-250	0.31	2<	1.4
Methyl-H-HCl-NIM-250	0.14	3.8	0.4
Methyl-C-HCl-MIM-200	1.73	3.7	2.2
Methyl-C-HCl-NIM-200	0.95	3.6	1.7
Methyl-H-HCl-MIM-200	0.37	2<	2.0
Methyl-H-HCl-NIM-200	0.18	4.2	0.7
Methyl-C-HCl-MIM-150	1.75	3.8	2.5
Methyl-C-HCl-NIM-150	1.01	3.4	1.2
Methyl-H-HCl-MIM-150	0.44	2<	3.0
Methyl-H-HCl-NIM-150	0.21	4.2	1.1

* Results obtained by the BET isotherm model; ** results obtained from the BJH desorption branch.

**Table 2:** Sorption isotherm model fitting and imprinting performance parameters of the all methyl-modified TiO_2_ microspheres.

Methyl-Modified TiO_2_ Microspheres	Model	Isotherm Parameters ^a^				*IF* (*q_max_*)	*α* (*q_max_*)
		*K* ^b^		*q_max_* (mg/g)			
		Bil	Pro	Bil	Pro		
Methyl-H-NIM-HCl	L-F	0.52 ± 0.03	0.55 ± 0.02	32 ± 2	39 ± 1	1.5	0.8
Methyl-H-MIM-HCl	H	11.30 ± 4.21	9.61 ± 5.32	49 ± 3	43 ± 5		1.1
Methyl-C-NIM-HCl	L-F	0.32 ± 0.03	0.33 ± 0.08	31 ± 2	46 ± 7	1.4	0.7
Methyl-C-MIM-HCl	H	9.61 ± 5.32	5.93 ± 2.31	43 ± 5	43 ± 5		1
Methyl-H-NIM-HCl-250	L-F	0.29 ± 0.03	0.33 ± 0.12	26 ± 3	15 ± 1	1.2	1.7
Methyl-H-MIM-HCl-250	H	13.23 ± 4.31	7.82 ± 2.53	30 ± 3	16 ± 2		1.9
Methyl-C-NIM-HCl-250	L-F	0.73 ± 0.03	0.43 ± 0.08	28 ± 1	25 ± 1	1.0	1.1
Methyl-C-MIM-HCl-250	H	2.11 ± 0.83	1.14 ± 0.75	28 ± 2	27 ± 3		1.0
Methyl-H-NIM-HCl-200	L-F	0.66 ± 0.03	0.40 ± 0.02	22 ± 2	14 ± 1	1.0	1.6
Methyl-H-MIM-HCl-200	H	2.53 ± 1.72	1.52 ± 0.64	23 ± 2	13 ± 1		1.8
Methyl-C-NIM-HCl-200	L-F	0.26 ± 0.02	0.60 ± 0.03	18 ± 1	21 ± 1	1.2	0.8
Methyl-C-MIM-HCl-200	H	1.91 ± 1.24	1.81 ± 1.25	22 ± 2	22 ± 2		1.0
Methyl-H-NIM-HCl-150	L-F	0.29 ± 0.03	0.36 ± 0.02	25 ± 3	16 ± 1	1.2	1.6
Methyl-H-MIM-HCl-150	H	5.92 ± 1.94	4.08 ± 2.22	30 ± 3	18 ± 2		1.7
Methyl-C-NIM-HCl-150	L-F	0.30 ± 0.06	0.22 ±0.02	21 ± 3	31 ± 1	1.1	0.7
Methyl-C-MIM-HCl-150	H	8.71 ± 2.82	6.10± 4.23	23 ± 2	39 ± 3		0.6
Methyl-H-NIM	L-F	0.40 ± 0.03	0.26 ± 0.03	32 ± 1	46 ± 4	1.8	0.7
Methyl-H-MIM	H	10.44 ± 3.22	9.51± 2.82	58 ± 4	52 ± 5		1.1
Methyl-C-NIM	L-F	0.38 ±0.02	0.13 ±0.04	37 ± 2	50 ± 9	1.4	0.7
Methyl-C-MIM	H	20.53 ± 5.43	10.51± 1.94	53 ± 5	44 ± 3		1.2

^a^ Non-linear fitting exhibiting *χ^2^* < 10^−7^; ^b^ (mol/L)^n^ for Hill isotherms; L/mg for L-F.

## Data Availability

Not applicable.

## Supplementary Materials

The following supporting information can be downloaded at: https://www.mdpi.com/article/10.3390/molecules27238510/s1, Figure S1: Equilibrium binding isotherms for the sorption of MIM bilirubin (●)/NIM bilirubin (●) and MIM protoporphyrin (●)/NIM protoporphyrin (●) by methyl-C-xxx-HCl-200 (a), methyl-C-xxx (b), methyl-C-xxx-HCl (c), methyl-H-xxx-HCl (d), methyl-C-xxx-HCl-250 (e), methylHx-HCl-250 (f), methyl-C-xxx-HCl-150 (g), and methyl-H-xxx-HCl-150 (h). Error bars represent the standard deviation of the mean result (*n* = 3).
