# Supplementary material for: Molecularly Imprinted Methyl-Modified Hollow TiO2 Microspheres
Source: Molecules. 2022 Dec 3;27(23):8510. doi: 10.3390/molecules27238510 (PMC9735797; doi:10.3390/molecules27238510)
Supplement: Supplementary file 1 [file molecules-27-08510-s001.zip › molecules-2021527-supplementary.pdf]

Molecularly imprinted methyl-modified hollow TiO_2 microspheres

Vanessa R. A. Ferreira, Manuel. A. Azenha *, Carlos M. Pereira and António F. Silva

CIQ-UP, Departamento de Química e Bioquímica, Faculdade de Ciências da Universidade do Porto, Rua do Campo Alegre, 4169-007 Porto, Portugal;
* Correspondence: mazenha@fc.up.pt; Tel.: +351-220402628

Supplementary Information

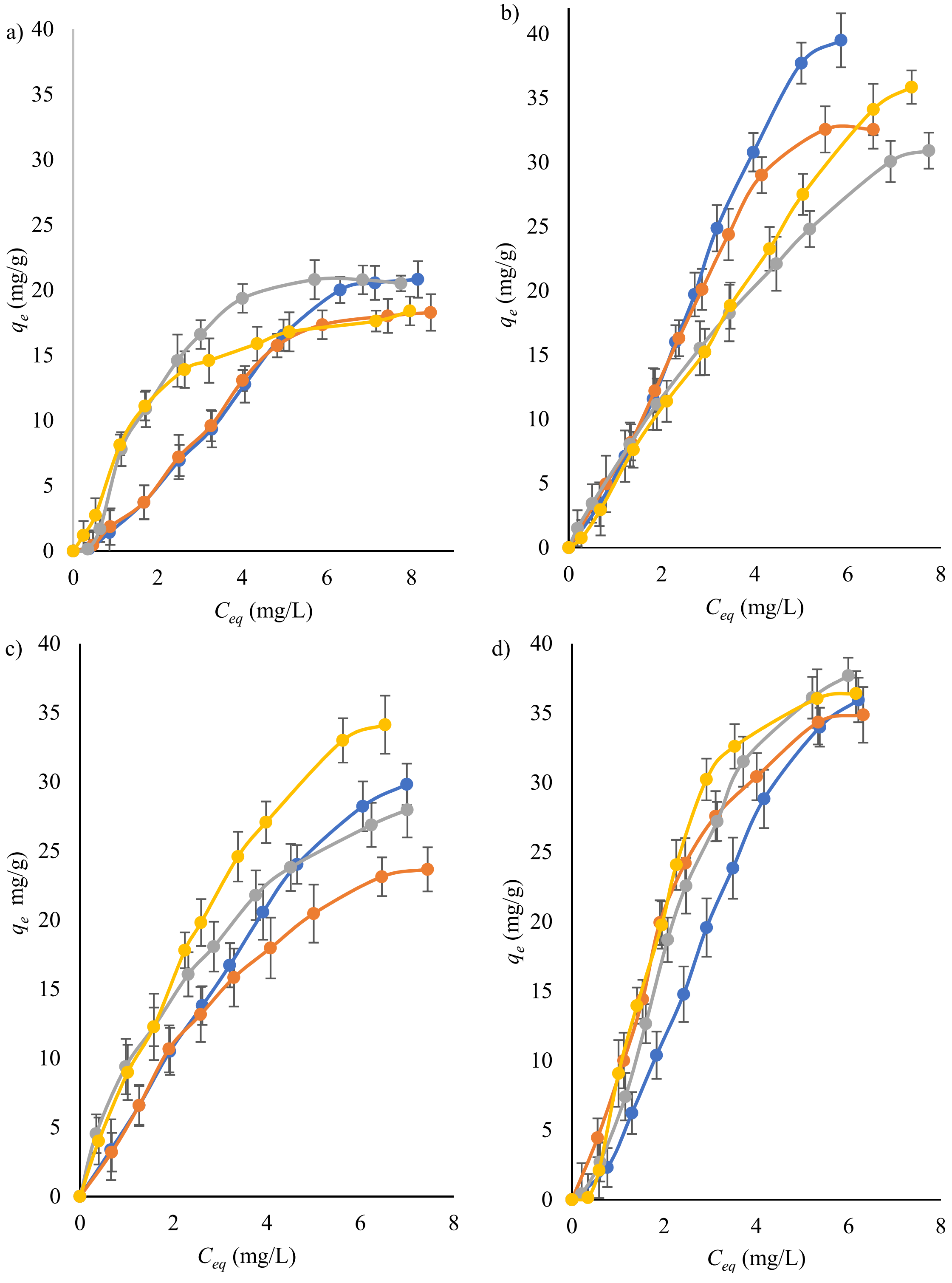

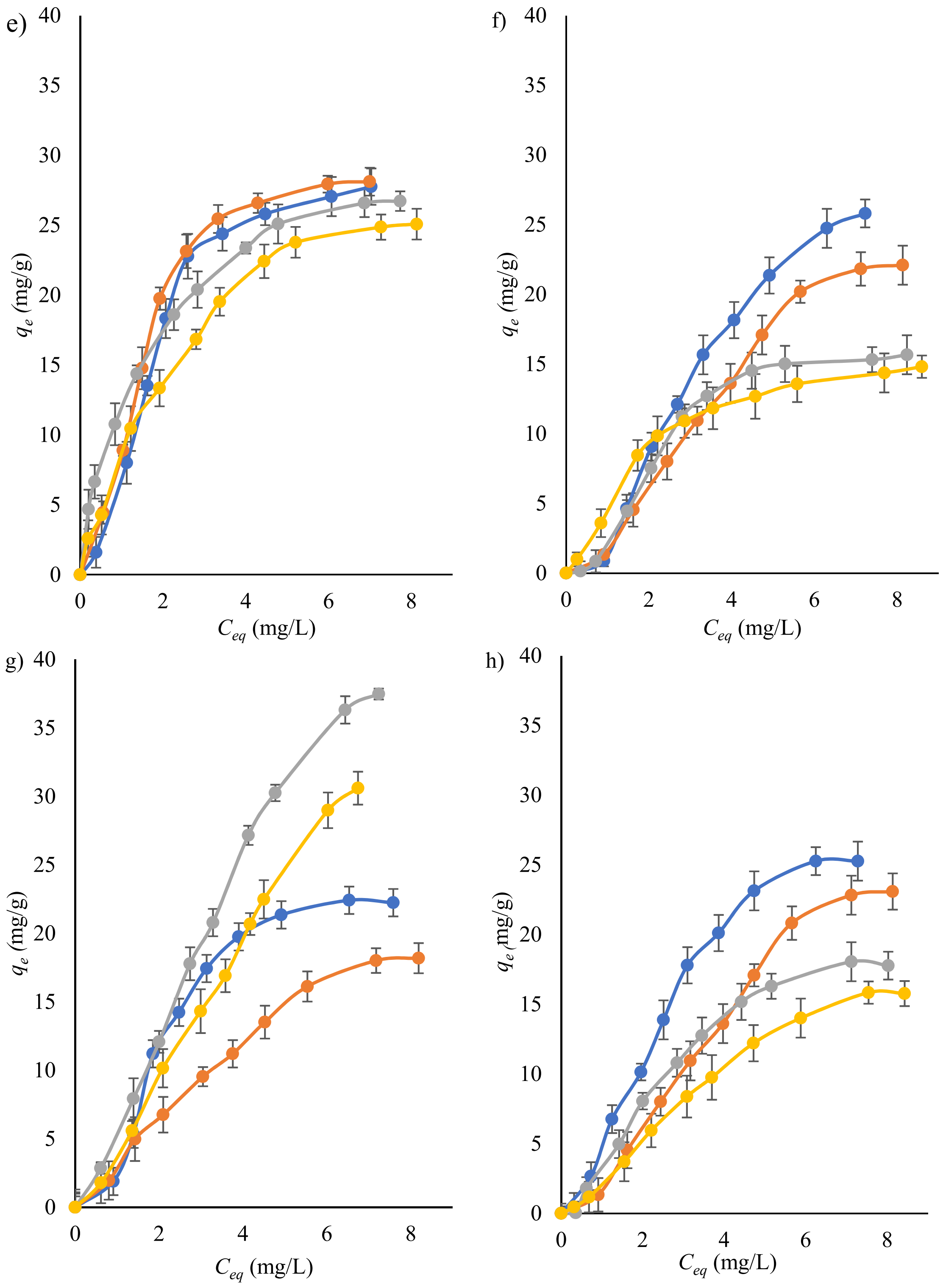

Figure S1. Equilibrium binding isotherms for the sorption of MIM bilirubin (●)/NIM bilirubin (●) and MIM protoporphirin (●)/NIM protoporphirin (●), by Methyl-C-xxx-HCl-200 (a); Methyl-C-xxx (b); Methyl-C-xxx-HCl (c); Methyl-H-xxx-HCl (d); Methyl-C-xxx-HCl-250 (e); MethylHx-HCl-250 (f); Methyl-C-xxx-HCl-150 (g); Methyl-H-xxx-HCl-150 (h). Error bars represent the standard deviation of the mean result (n=3).
